# Advancing pancreatic cancer research and therapeutics: the transformative role of organoid technology

**DOI:** 10.1038/s12276-024-01378-w

**Published:** 2025-01-16

**Authors:** Jihao Xu, Minh Duc Pham, Vincenzo Corbo, Mariano Ponz-Sarvise, Tobiloba Oni, Daniel Öhlund, Chang-Il Hwang

**Affiliations:** 1 Department of Microbiology and Molecular Genetics, University of California, Davis, Davis, CA 95616 USA; 2 Department of Engineering for Innovation Medicine, University of Verona, Verona, Italy; 3 Department of Medical Oncology and Program in Solid Tumors, Cima-Universidad de Navarra, Cancer Center Clinica Universidad de Navarra (CCUN), Pamplona, Pamplona, Spain; 4 Whitehead Institute for Biomedical Research, Cambridge, MA USA; 5 Umeå University, Department of Diagnostics and Intervention, and Wallenberg Centre for Molecular Medicine at Umeå University, Umeå, Sweden; 6 University of California Davis Comprehensive Cancer Center, Sacramento, CA 95817 USA

**Keywords:** Cancer models, Experimental models of disease

## Abstract

Research on pancreatic cancer has transformed with the advent of organoid technology, providing a better platform that closely mimics cancer biology in vivo. This review highlights the critical advancements facilitated by pancreatic organoid models in understanding disease progression, evaluating therapeutic responses, and identifying biomarkers. These three-dimensional cultures enable the proper recapitulation of the cellular architecture and genetic makeup of the original tumors, providing insights into the complex molecular and cellular dynamics at various stages of pancreatic ductal adenocarcinoma (PDAC). We explore the applications of pancreatic organoids in dissecting the tumor microenvironment (TME); elucidating cancer progression, metastasis, and drug resistance mechanisms; and personalizing therapeutic strategies. By overcoming the limitations of traditional 2D cultures and animal models, the use of pancreatic organoids has significantly accelerated translational research, which is promising for improving diagnostic and therapeutic approaches in clinical settings, ultimately aiming to improve the outcomes of patients with pancreatic cancer.

## Pancreatic Organoids Transform Cancer Research and Treatment

Pancreatic cancer is a challenging disease to study and treat. This article discusses how researchers have developed pancreatic organoids to better study this cancer. Organoids are created by growing cells in a specialized 3D matrix, allowing them to form structures that resemble tissues found in the body. This method is more effective than traditional 2D cultures because it better replicates the natural environment of the cells. Researchers use these organoids to study cancer progression, test new drugs, and understand genetic changes in tumors. They can be made from small tissue samples, making them useful for studying advanced cancer stages where tissue is scarce. The findings from organoid studies help identify potential new treatments and improve our understanding of pancreatic cancer biology. In conclusion, pancreatic organoids offer a promising tool for advancing cancer research and developing personalized treatments.

This summary was initially drafted using artificial intelligence, then revised and fact-checked by the author.

## Introduction

Pancreatic organoids represent one of the most recent and impactful advancements in pancreatic cancer research, offering a sophisticated model that has been widely adopted in various research settings. The Tuveson and Clevers laboratories developed murine and human models of pancreatic ductal adenocarcinoma (PDAC) progression using a three-dimensional tissue culture technique known as pancreatic organoids^1^. A pancreatic organoid is defined as a group of adult and differentiated pancreatic epithelial cells organized into a 3D spheroid that can be expanded long-term ex vivo with a defined cellular organization, including basal‒apical polarity and lumen formation^2^. A spheroid is defined as a three-dimensional cell aggregate without predefined culture conditions, and an organoid is defined as a 3D cellular structure that mimics key aspects of an organ’s architecture and function, which is formed through the self-organization of stem cells, progenitors, or differentiated cells, relying on cell‒cell and cell‒matrix interactions to create a simplified yet functional model of the original tissue^3^. Unlike traditional 2D cell line or xenograft transplantation models, pancreatic organoids derived from a small quantity of both murine and human tissues better recapitulate the physiologically relevant characteristics of PDAC progression in vitro, can be generated more efficiently in a relatively short time frame, and allow PDAC stage-specific comparisons via genetic, epigenetic, transcriptomic, and proteomic profiling^1,4,5^, thereby facilitating the discovery of novel biomarkers and therapeutic strategies.

The journey to developing effective pancreatic organoid cultures began with the first work of the Ruby laboratory in 1980, which marked the genesis of pancreatic 3D culture by achieving successful ex vivo culture of the ductal epithelium from the rat pancreas^6^. In 2012, the Bar-Sagi laboratory introduced a method enabling the passaging and expansion of murine primary ductal epithelial cells into spheroids^7^. This technique involves the microdissection of murine pancreatic ducts, enzymatic dissociation of the ducts into single cells or small clusters, and plating in an 80% Matrigel matrix, which results in the formation of spheroids within 48 hours^7^. However, these approaches are limited in terms of their capacity for cell expansion.

The Ku and Riggs laboratories introduced a method employing a 1% methylcellulose and 5% Matrigel mix, facilitating the formation of “ring” colonies characterized by hollow spheres with a ductal epithelial identity^8^. Notably, this culture system selectively supported the proliferation of ductal cells while excluding cells with endocrine or acinar identities^8^. Furthermore, when the cells were placed into a laminin-based matrix, the cultured cells could differentiate into endocrine/acinar cells^8^, indicating the existence of potential pancreatic progenitor cells in the populations. The Rustgi laboratory also reported the possibility of growing spheroids from adult and embryonic mouse pancreata, as well as from a genetically engineered mouse model (GEMM) harboring *Kras*^*+/LSL-G12D*^; *Trp53*^*+/LSL-R172H*^; *Pdx1-Cre* (KPC) alleles^9^. The laboratory developed a method utilizing *Dolichos biflorus* agglutinin lectin, a glycoprotein that specifically recognizes pancreatic ducts, to isolate pancreatic ductal cells selectively^8^. Culturing these cells in type I collagen resulted in the formation of single-cell-layered spheroids with hollow cores within ten days^9^.

Building on their prior success with intestinal organoids, where the use of Wnt ligands and growth factors enabled the growth and maintenance of the mini-gut, the Clevers laboratory applied similar principles to pancreatic tissues. Given that pancreatic progenitor cells also require Wnt signaling for their growth, a murine pancreatic organoid culture method was developed. This method involves embedding fragmented normal murine pancreatic ducts in a Matrigel matrix supplemented with roof plate-specific spondin 1 (RSPO1), a Wnt agonist^4^. This process leads to the formation of single-cell-layered organoids containing adult pancreatic progenitor cells with hollow cores within 24–48 h, which are capable of maintaining long-term expansion, passaging, and bipotency without malignant transformation for more than ten months. Intriguingly, upon orthotopic transplantation into the mouse pancreas, these organoids generate ductal structures, indicating the preservation of the biological characteristics inherent to the ductal nature of the organoid culture method. Using a similar methodology, the Kuo laboratory developed an air‒liquid interface organoid culture model in which minced pancreatic ductal tissues from wild-type or genetically engineered mice with *Kras*^*+/LSL-G12D*^; *Trp53*^*flox/flox*^ alleles embedded in a collagen matrix were exposed to both culture media and air^5^. These organoids comprised epithelial, endocrine, and stromal components and demonstrated oncogenic transformation potential in vitro upon the addition of an adenovirus expressing Cre recombinase, enabling long-term expansion and passaging of oncologically transformed organoids in contrast to the limited passaging of wild-type or untransformed pancreatic ductal organoids.

**Fig. 1:**
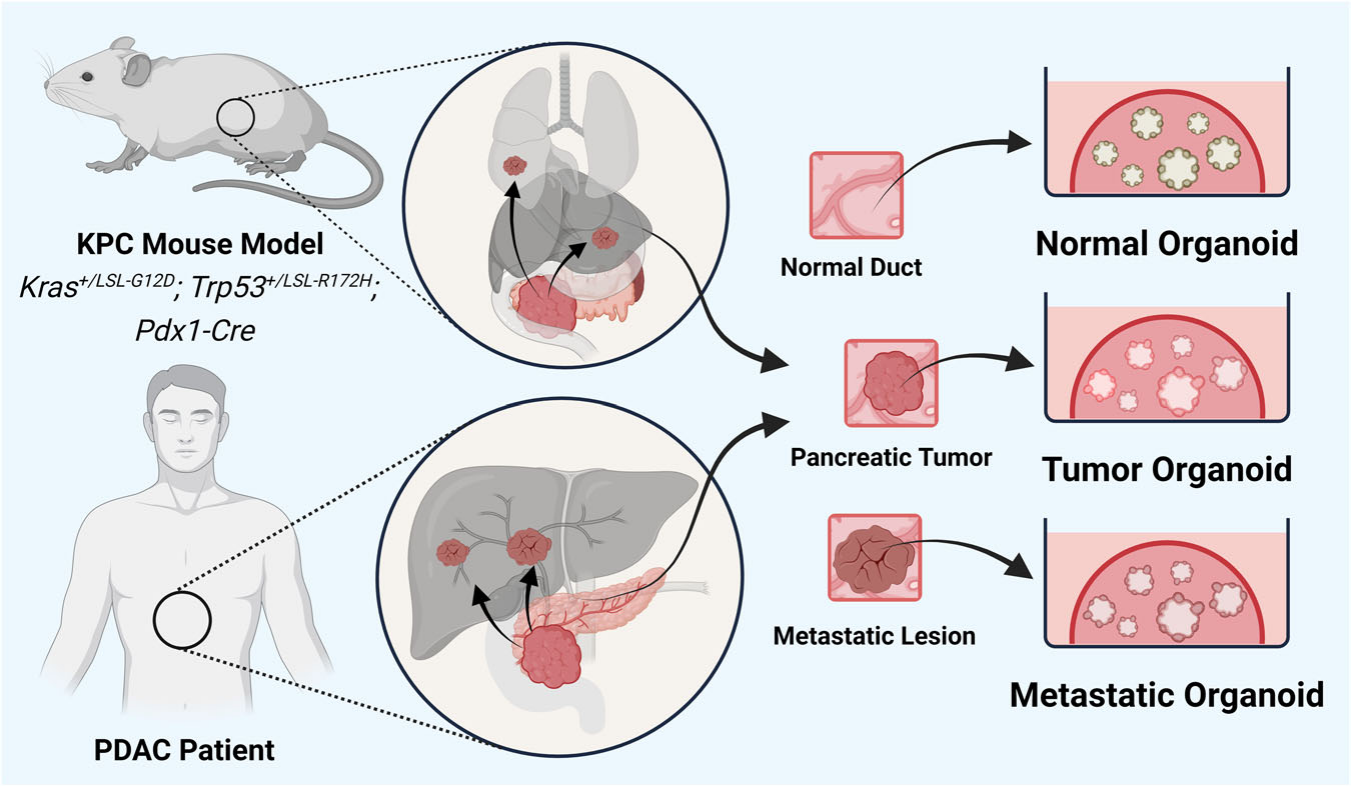
Organoid models of pancreatic cancer. Pancreatic organoid cultures can be established from tumors and metastatic lesions of genetically engineered PDAC mouse models and PDAC patients. The culture conditions allow normal and preneoplastic epithelial cells to grow for comparison. Notably, neoplastic cells from a small amount of tissue, such as that obtained from fine needle aspiration, can be expanded for research purposes.

Expanding upon their foundational work in organoid technology, the Tuveson and Clevers laboratories further refined robust pancreatic organoid models to recapitulate various stages of pancreatic cancer progression in both mice and humans^1^. Using a Matrigel matrix with defined concentrations of various growth factors and Wnt agonists, such as RSPO1 and WNT3, they developed culture conditions that robustly expand neoplastic pancreatic populations, as well as normal pancreatic ductal epithelial cells (Fig. 1). While normal pancreatic ductal cells are typically quiescent and exhibit limited proliferation in vivo, in organoid cultures, these cells –particularly human normal pancreatic ductal organoids - proliferate at a similar rate to neoplastic cells, with a limited expansion capability but no accumulation of mutations^1^. This discrepancy highlights a significant difference between in vitro and in vivo conditions but also provides a robust platform for comparative studies. Organoid technology enables researchers to use these proliferating normal cells as a dynamic control to compare with neoplastic cells, offering a unique opportunity to study pancreatic cancer progression at the molecular and cellular levels in a controlled, reproducible environment. This model system thus remains invaluable for dissecting the complexities of pancreatic cancer and developing targeted therapeutic strategies^1^.

One of the critical advantages of pancreatic organoid models is their ability to be established from very small tissue samples, such as those obtained through fine needle aspiration (FNA). This property is particularly relevant in the context of PDAC, where most patients are diagnosed at an advanced stage and are ineligible for surgical resection, limiting the availability of tissue samples for research. For example, the majority of PDAC samples in The Cancer Genome Atlas (TCGA)-PAAD are derived from patients with early-stage tumors, potentially skewing data representation toward these early-stage patients. Moreover, PDAC is known for its extensive desmoplastic reaction, leading to a low signal-to-noise ratio that complicates molecular and cellular analyses. Organoid models, therefore, provide a unique and powerful tool for capturing and studying the biology of various stages of PDAC. Compared with traditional 2D cultures and xenograft models, pancreatic organoids represent a significant advancement in the study of PDAC, offering a more physiologically relevant and efficient model. By enabling detailed genetic and molecular analyses at various stages of disease progression, these organoids provide a powerful platform for identifying novel biomarkers and therapeutic targets. The evolution of 3D culture techniques, as demonstrated by various pioneering laboratories, has greatly enhanced our ability to study pancreatic cancer, paving the way for more effective and targeted treatments in the future.

## Molecular and cellular characteristics of pancreatic organoid cultures

In this review, we focus primarily on the pancreatic organoid models developed by the Tuveson and Clevers laboratories, although various methodologies to grow pancreatic cells as organoids exist. Understanding the differences between 2D and 3D culture models is important for choosing proper preclinical models that align with our research questions. Although 2D cell culture plays a pivotal role in cancer research, it often fails to recapitulate the complexities of in vivo biology, leading to challenges in translating research findings to clinical applications^10^. This challenge is largely due to the lack of an appropriate cellular organization in 2D cell cultures, which affects downstream cell signaling and functions^11^. For example, when 2D-adapted PANC-1 cells were plated in a methylcellulose-based matrix, upregulation of the expression of key markers associated with hypoxia and anaerobic glycolysis, including glucose transporter 1, lactate dehydrogenase, and HIF-1a, was observed as the cells formed spheroids, indicating hypoxia and aerobic glycolysis^12^. Moreover, an analysis of the extracellular matrix revealed increased expression of collagen I and fibronectin I, along with chemoresistance-related genes, conferring gemcitabine resistance in vitro^12^. Changes in biophysical properties between 2D and 3D cultures might significantly affect the cellular organization, subsequently impacting cell signaling and molecular profiles, such as transcriptional, metabolomic, and proteomic landscapes.

Unlike 2D cultures, which often fail to maintain epithelial characteristics, 3D cultures tend to better mimic the structural and histopathological features of pancreatic tissues in vivo. Multiple studies have reported that in pancreatic 3D cultures, pancreatic epithelial cells are organized with the proper basal‒apical polarity and cytoskeletal establishment observed in vivo, as evidenced by the localization of microvilli, transmembrane glycoprotein mucin 1 (MUC1), tight junctions, and F-actin at the lumen-facing apical surface, and lamina and basement membrane collagen localize to the basal surface^6,8,9^. Moreover, organoids derived from pancreatic intraepithelial neoplasia (PanIN) recapitulated the in vivo histopathology, with enlarged pleomorphic nuclei and cribriform formations^5^. Overall, the pancreatic organoid model accurately recapitulates the tissue organization observed in vivo, indicating its reliability as a research model for studying pancreatic progression.

Patient-derived organoids (PDOs) have also been shown to replicate several in vivo neoplastic cell states^13–15^. This property is extremely relevant considering that the differing sensitivities displayed by different transcriptional cell states often coexist within the same tumor mass. In vivo pancreatic cell states are shaped by the integration of cell-intrinsic and cell-extrinsic inputs. A fact that is less understood is the relative contributions of genetic and microenvironmental factors to those cell states. Proper modeling of PDAC molecular phenotypes and the subsequent therapeutic responses would therefore require a culture system that allows (i) the capture of the somatic genetic and interindividual variations and (ii) the emergence of microenvironment-instructed cell states. PDOs can be reliably established from a large population of PDAC patients, including those with reduced availability of starting material (i.e., locally advanced or metastatic disease), and have been shown to properly capture the genetics of the original tissue^16,17^. Moreover, in vivo transcriptional cell states, including those associated with therapy resistance^14,15^, can be observed in PDOs exposed to either tumor microenvironment (TME) cues or pharmacological compounds.

Therefore, this dependency of PDO transcriptional phenotypes on the specific microenvironment to which they are exposed can be considered both a benefit and a limitation. For example, stiff matrix conditions, as observed in PDAC, can induce the epithelial‒mesenchymal transition (EMT) and promote chemoresistance by enhancing cellular mechanotransduction and altering cellular signaling pathways^18,19^.

Pancreatic organoid models have been proven valuable for recapitulating disease progression in vivo and patient settings. In murine pancreatic organoids, notable upregulation of Muc5ac, Muc6, and Tff1 was observed in murine PanIN (mP) organoids, indicative of preserved PanIN features^1^. These organoids exhibited the ability to form PanIN lesions characterized by increased cell proliferation and stroma formation when orthotopically transplanted into the mouse pancreas. In addition, differentially expressed genes were validated in murine tumor-derived organoids and confirmed in both mouse PanINs and tumors, as well as human pancreatic tissues, highlighting the robust utility of organoid models for mimicking in vivo conditions and advancing our discovery of potential biomarkers. Both transplanted murine PDAC tumor (mT) and metastatic (mM) organoids exhibited stroma formation and resembled autochthonous tumors from the KPC mouse model. In contrast to mT organoids, which required several months of progression to metastasis upon orthotopic transplantation, mM organoids metastasized within one month. This striking difference in metastatic potential highlights the value of organoid models in studying cancer metastasis mechanisms and has facilitated a number of discoveries related to the epigenetic mechanisms driving these processes^20–22^. Regarding clinical relevance in patients, distinct morphological and molecular differences were observed in human normal (hN) and tumor (hT) (including human fine needle biopsy samples, hFNAs) organoids. hN organoids displayed a cuboidal morphology, whereas hT organoids displayed a dysplastic tall columnar morphology. Furthermore, hT organoids were highly aneuploid and harbored mutations in KRAS, TP53, SMAD4, and CDKN2A, along with MYC amplification and the loss of the tumor suppressors TGFBR2 and DCC. Upon orthotopic transplantation into Nu/Nu mice, hN organoids formed normal ducts, whereas hT organoids rapidly progressed to form PanIN-like lesions within one month. These animals subsequently develop infiltrative carcinoma with a prominent desmoplastic reaction^1^.

Together, these findings suggest that pancreatic organoid culture provides a physiologically relevant platform for studying pancreatic cancer biology and exploring potential therapeutic strategies. By recapitulating key molecular and cellular characteristics of the disease, organoid models provide valuable insights into disease mechanisms and offer opportunities for personalized medicine approaches. Continued research in this area promises to further improve our understanding of pancreatic cancer and improve patient outcomes.

## Organoid models of transcriptional and epigenetic dynamics

Pancreatic carcinogenesis is driven mostly by gain-of-function mutations in the oncogene KRAS, which cooperate with loss-of-function mutations in tumor suppressor genes such as TP53, CDKN2A, and SMAD4^23^. However, recurrent genetic mutations that further drive cancer aggressiveness and metastatic capabilities remain largely unknown. Since metastatic PDAC often has a mutation landscape similar to that of the primary tumor^24^, comprehending cell-autonomous factors, such as transcription factor (TF)-directed programming and epigenetic alterations, driving PDAC progression and metastasis is essential for the development of effective cancer therapeutics. PDO models of tumor and metastatic tissues represent promising platforms that address many of these limitations^25^. These three-dimensional cultures maintain the epigenetic and transcriptional status of the original tumors^1,20,25^, including basal-like and squamous characteristics, and allow for uniform culture conditions and longitudinal sampling. Unlike 2D cultures, which often lead to highly aggressive and metastatic phenotypes^26^, organoids better preserve the heterogeneity and behavior of the original tumor cells^1^, providing a more accurate representation of cancer cells. A significant advantage of organoid models is their ability to be derived from fine needle biopsy samples, enabling the expansion of organoids for various experimental approaches, including multiomics studies^16,27^. This property is particularly noteworthy given that most omics experiments with human PDAC samples have been performed on early-stage tumors from surgical resections^28^. Here, we comprehensively review how PDAC organoid models improve our understanding of how epigenetic alterations promote PDAC progression and metastasis.

Recent studies using PDAC organoid models have underscored the pivotal role of TFs in driving epigenetic alterations that contribute to pancreatic cancer progression. Notably, FOXA1 plays a critical role in reprogramming enhancer landscapes to drive metastatic phenotypes in PDAC. Briefly, FOXA1 activates foregut developmental gene programs, thereby promoting metastasis. This phenomenon was elucidated using paired tumor- and metastasis-derived organoids derived from GEMMs of pancreatic cancer, which provided a robust platform for studying these epigenetic alterations^20^. Similarly, we recently identified Engrailed-1 (EN1) as a crucial mediator that enhances the aggressive behavior and metastatic potential of PDAC cells, primarily through its epigenetic regulation of target genes involved in cell death/survival^22^. The TF ecotropic viral integration site 1 (EVI1), known for its role in hematopoietic malignancies, has also been implicated in PDAC. EVI1 activates superenhancers and leads to the upregulation of genes that drive tumorigenesis and metastasis, highlighting its role in promoting the aggressive nature of PDAC^21^. Furthermore, TEAD2 has been shown to facilitate the transition of PDAC cells to a basal-like subtype characterized by increased angiogenesis and metastatic ability through the activation of endothelial-like enhancers^29^. These findings collectively illustrate how aberrantly expressed TFs can reprogram the epigenome, resulting in increased aggressiveness and metastatic potential in pancreatic cancer, suggesting new avenues for therapeutic interventions targeting these epigenetic changes.

The utilization of PDAC organoids to study DNA methylation and its role in PDAC progression has provided significant insights. Previously, Bailey et al. identified the squamous subtype of PDAC, characterized by hypermethylation and concordant downregulation of genes that govern pancreatic endodermal cell fate determination, including PDX1, MNX1, GATA6, and HNF1B, leading to a complete loss of endodermal identity^30^. This discovery underscores the critical role of DNA methylation in defining the molecular subtypes of PDAC and their respective prognoses. Building on these findings, our research utilizing whole-genome bisulfite sequencing (WGBS) in both mouse and human PDAC organoid models identified stage-specific and subtype-specific DNA methylation signatures^31^. Notably, the squamous subtype displayed hypermethylation of GATA6-binding sites, highlighting a significant epigenetic shift that silences progenitor subtype-associated genes and enforces the squamous phenotype. These findings reveal the significant impact of DNA methylation on PDAC progression and molecular subtype differentiation. The ability to use organoids to study these epigenetic alterations not only improves our understanding of the molecular underpinnings of PDAC but also has potential for developing diagnostic markers for molecular subtypes.

In conclusion, PDAC organoid models have emerged as transformative tools in pancreatic cancer research, enabling a more nuanced understanding of the molecular and epigenetic alterations associated with this disease. These models offer distinct advantages over traditional preclinical models, preserving the complexity and heterogeneity of the original tumors. The ability to derive organoids from fine needle biopsy samples allows for longitudinal studies of tumor progression and metastasis, including advanced stages of PDAC. This capability is crucial for investigating epigenetic alterations, such as DNA methylation and TF-driven enhancer reprogramming, which play pivotal roles in PDAC aggressiveness and metastasis. Research utilizing these organoids has highlighted the contributions of key TFs, such as FOXA1, EN1, EVI1, and TEAD2, in driving epigenetic changes that enhance tumor malignancy. Additionally, identifying subtype-specific DNA methylation patterns, particularly in the squamous subtype, underscores the potential of organoids in developing diagnostic markers and targeted therapies. As we continue to leverage the power of organoid technology, we anticipate significant advancements in our fundamental understanding of pancreatic cancer and the development of innovative clinical interventions to improve patient outcomes.

## Organoid models for the development of therapeutics

**Fig. 2:**
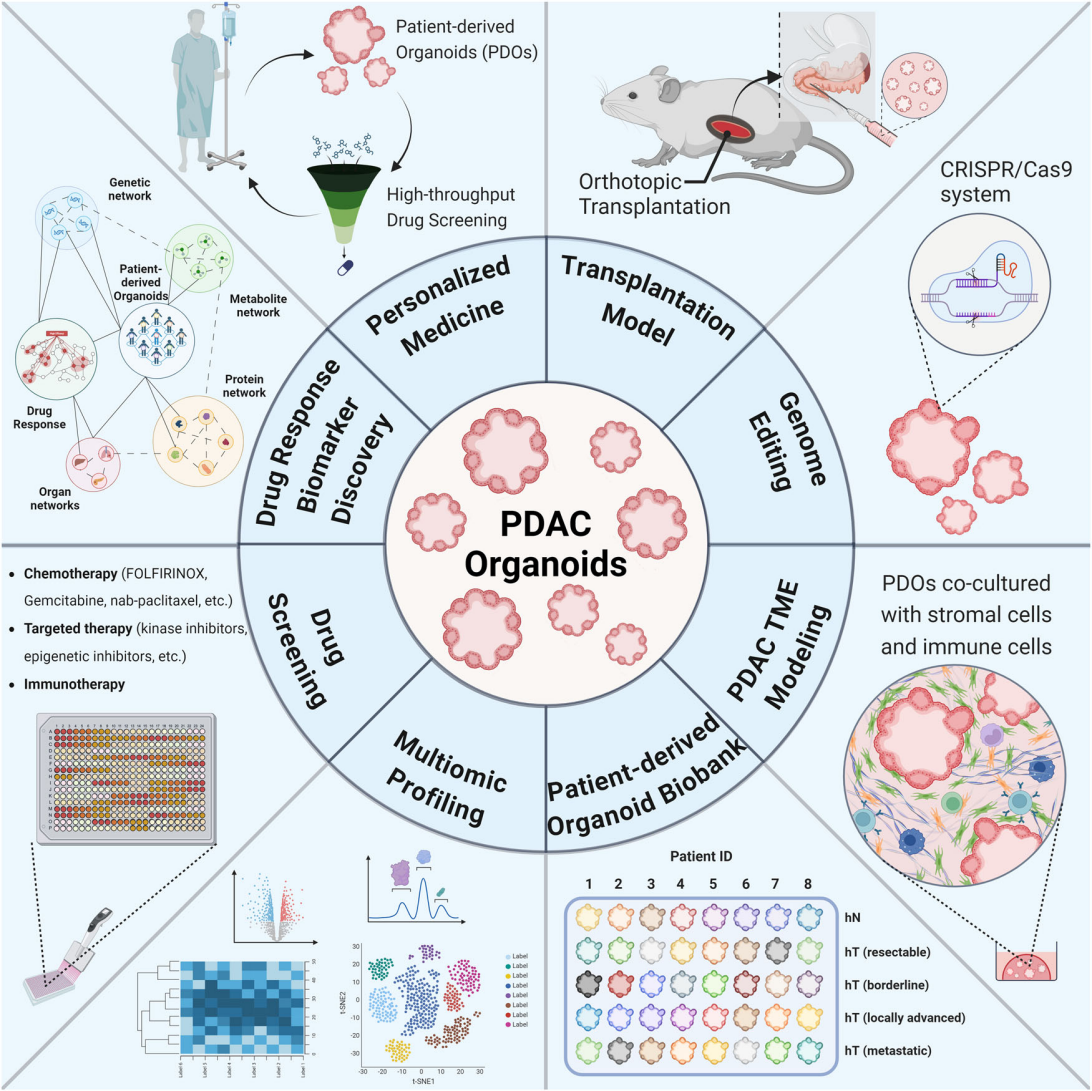
Applications of PDAC organoids. PDAC organoids are currently used for a variety of different research purposes. These applications include, but are not limited to, personalized medicine, therapeutics, studies of drug resistance mechanisms, drug/genetic screening, various omics approaches, biobanking, and preclinical models.

In the clinical treatment of PDAC, oncologists face several challenges. Current standard-of-care therapies such as FOLFIRINOX or gemcitabine in combination with nab-paclitaxel and 5-FU combined with irinotecan and oxaliplatin (FOLFIRINOX) often achieve only modest improvements in patient survival^32,33^. These outcomes can largely be attributed to the complex pancreatic TME, which significantly hinders effective drug delivery and distribution^34^. Additionally, while GEMMs are widely used in preclinical settings for drug testing, robust testing of these models often proves infeasible^35^. This finding highlights a critical gap in our ability to translate chemotherapeutic advances into meaningful clinical benefits. Drawing inspiration from microbiology, where the Kirby–Bauer disk diffusion test enables precise antibiotic selection by testing various agents against patient-isolated bacteria, cancer researchers are exploring similar strategies. The use of organoid models derived from patient tumors, therefore, is a promising approach. These organoids facilitate the screening of multiple therapeutic agents to determine the most effective treatment for an individual’s cancer cells (Fig. 2). Although organoid models still face challenges, such as long turnaround times, albeit shorter than those of patient-derived xenograft models, they represent a significant step toward personalized and effective cancer therapy by mimicking patient-specific tumor biology more accurately than other preclinical models.

In 2018, Tiriac et al. provided important insights into the predictive power of organoids by demonstrating that drug responses in PDOs reflect the clinical responses observed in patients^25^. This correlation confirms the utility of organoids in personalizing treatment regimens, potentially revolutionizing the approach to pancreatic cancer therapy by allowing for tailored treatments based on the predicted efficacy and resistance profiles observed in the organoids. In particular, PDOs established during the clinical course of an individual patient successfully reproduced the development of resistance to all commonly used chemotherapies for PDAC. Another pilot trial involving 76 patients by Hidalgo’s group, namely, the HOPE trial (Harnessing Organoids for Personalized Therapy), also corroborated the use of PDOs in recapitulating patients’ drug responses^36^. In a similar retrospective study of 136 samples from both surgical resections and fine needle aspirations/biopsies, Demyan and colleagues reported the promising ability of PDOs to predict the response to neoadjuvant chemotherapy^37^. Additionally, the authors highlighted the feasibility of rapid drug screening using PDOs with turnaround data at a minimum of only 7 days after tissue resection. This fast generation of PDO drug screening, if further validated, could be a milestone in widely applying PDO technology in personalized neoadjuvant chemotherapy for PDAC patients. In the latest prospective study published in 2024 by Jaulin’s group, PDOs from 34 PDAC patients with complete full clinical follow-up were compared to patient responses to a panel of 25 approved antitumor therapies (chemograms)^38^. This study revealed the robust predictive value of PDOs in the clinical setting, with a chemogram sensitivity of 83.3% and specificity of 92.9%. Moreover, patients receiving “hit” treatments identified by PDO drug testing had significantly higher overall responses and progression-free survival rates. These studies highlight the potential of organoids not only in advancing our understanding of PDAC at the molecular level but also in enhancing the precision of treatment regimens tailored to individual patient profiles. The use of organoids allows for a more dynamic assessment of drug responses, potentially leading to more effective and sustainable therapeutic outcomes.

Not surprisingly, PDOs have been extensively used as drug screening platforms to identify novel targets and resistance mechanisms for PDAC. In an effort to establish a biobank of 30 PDO lines, Clevers’s group reported the application of PDOs in identifying novel therapeutic agents that had not yet been exploited in the clinic^39^. They reported that PDOs with elevated MTA levels rather than mutation of MTAP were responsive to EZP01556, an inhibitor of the protein arginine methyltransferase 5 (PRMT5). These findings also suggest that the level of MTA might be a better marker for predicting the response to PRMT5 inhibitors. Similarly, by performing CRISPR-Cas9 genome editing and drug screening on a biobank of 31 PDOs, Schwank’s group provided preliminary results supporting the efficacy of emetine and ouabain in PDAC treatment through interference with survival under hypoxia^40^. The authors also elucidated the association between missense mutations in *ARID1A* and increased sensitivity of PDAC to kinase inhibitors (dasatinib and VE-821). Moreover, Huang and colleagues subjected PDOs to epigenetic inhibitors and revealed the sensitivity of certain PDOs to UNC1999, which targets the histone modifier EZH2^41^. These findings suggest the potential of EZH2 inhibition in suppressing the proliferation of tumors, especially those resistant to gemcitabine. Zhou and colleagues, with a similar goal of finding potential drugs to overcome gemcitabine resistance in PDAC, identified irbesartan in a screen of 1304 FDA-approved compounds on a PDO biobank^42^. As an angiotensin type 1 antagonist, irbesartan reportedly confers resistance by inhibiting the Hippo/YAP1 pathway and reducing the expression of c-Jun, which can upregulate genes involved in stemness maintenance and iron metabolism. Gulay et al. utilized PDOs to show that treatment with MRTX1133, a specific KRAS^G12D^ inhibitor, was effective at low concentrations but eventually resulted in resistance due to the upregulation of EGFR and HER2. Thus, this study revealed a synergistic effect of inhibiting both KRAS and ERBB signaling to circumvent acquired resistance in MRTX1333-treated patients^43^. In addition, we previously provided critical insights into the resistance mechanisms that undermine targeted therapies. MEK and AKT inhibitors, while initially effective, inadvertently activate compensatory RTK pathways, specifically ERBB2 and ERBB3, leading to drug resistance^44^.

Additionally, PDOs allow multiomic analyses of patient samples at a large scale and thus facilitate the discovery of distinct markers for predicting drug responses. In a comprehensive approach to understand the link between chromatin accessibility and drug sensitivity, Shi et al. performed a drug screen of a total of 283 chemicals targeting epigenetic-related signaling pathways on 35 PDOs^27^. By integrating ATAC-seq and transcriptome profiles into drug response data, they suggested that chromatin accessibility signatures can predict the chemosensitivity of PDAC and reveal potential underlying mechanisms. For example, an ATAC-seq peak on chr10 is significantly associated with resistance to 5FU and paclitaxel and is positively correlated with *BAG3* expression. Interestingly, the resistance of PDAC to chemotherapy can even be associated with its metabolism, which was reported in a study by the Jin laboratory. By characterizing the metabolomic profiles of 28 established PDOs, this study revealed two metabolic subtypes of PDAC with prognostic significance and therapeutic implications^45^. While the lipomet-PDAC group, which has high lipid metabolism, is more sensitive to chemotherapy, the glucomet-PDAC group, which relies on carbohydrate and nucleotide metabolism, is more resistant to chemotherapy and is associated with a poor prognosis. This resistance can be leveraged by pharmacologically targeting the GLUT1/ALDOB/G6PD axis.

In conclusion, the utilization of organoid models of pancreatic cancer represents a significant advancement in studies of cancer therapeutics and resistance mechanisms. These three-dimensional cultures offer an unprecedented opportunity to tailor and refine therapeutic strategies for individual patients. By mirroring patient-specific contexts, organoids provide more accurate predictions of drug efficacy and resistance patterns, facilitating the development of more effective combination therapies. This approach would facilitate personalized/precision medicine in PDAC therapeutics, ultimately improving patient outcomes by aligning the therapeutic interventions more closely with the unique characteristics of each tumor.

## Organoid Models of the PDAC Microenvironment

The dense stroma, which is primarily composed of activated cancer-associated fibroblasts (CAFs) and a deposited collagen-rich extracellular matrix (ECM), is a hallmark of PDAC and presents significant challenges in drug delivery, as outlined in the seminal work by Olive et al. ^34^. The dense ECM impedes the effective penetration of therapeutic agents. Various studies targeting CAFs in murine models, such as stromal depletion via Hedgehog signaling^34^ or depletion of the ECM component glycosaminoglycan hyaluronan^46^, have shown promising results in enhancing chemotherapeutic delivery. However, the clinical translation of CAF-targeting strategies has yet to yield significant improvements in the overall survival or progression-free survival of patients with PDAC^47,48^. A subsequent mechanistic study revealed that the inhibition of Hedgehog signaling induced an immunosuppressive TME by promoting CAF subtype differentiation^49^, emphasizing the need for improved preclinical tools to decipher PDAC stromal biology. In addition, PDAC is often characterized as a “cold” tumor that is largely resistant to immunotherapeutic interventions due to the minimal presence and activity of immune effector cells within the tumor mass^50^. Addressing these complexities requires innovative models that can accurately replicate the intricate interactions within the TME. The recent development of coculture models with organoids and their respective stromal cell types, which encompass the diverse cellular composition of PDAC, including CAFs, immune cells, and tumor cells, represents a promising platform. Coculture models of organoids and various cell types from the PDAC TME provide a comprehensive approach for examining the dynamic interplay between cell types. Additionally, these models are invaluable for developing and testing new strategies aimed at enhancing drug delivery and combating the immune-excluded nature of PDAC.

Through the development of coculture models combining PDAC organoids and pancreatic stellate cells (PSCs), which are considered major precursors to CAFs in the pancreas, the Tuveson laboratory provided a foundation for understanding the intricate TME in PDAC. The initial study by Öhlund et al. revealed mutual benefits in which both organoids and PSCs promoted the other’s growth, activated the transformation of PSCs into CAFs, and produced the dense ECM typical of PDAC^51^. Encouraged by these findings, we explored the effects of conditioned media from PDAC organoids on quiescent PSCs. In contrast to our expectations, the expression of alpha smooth muscle actin (αSMA), a previously widely used marker for CAFs, was dramatically suppressed in a subset of activated PSCs, revealing a different subtype of CAFs in which the expression of cytokines was induced instead. This unexpected outcome prompted further investigation into the identification of additional CAF subtypes in addition to classical myofibroblasts (myCAFs). Briefly, the transformation of PSCs into myCAFs is characterized by high αSMA expression, whereas inflammatory CAFs (iCAFs) are characterized by elevated levels of inflammatory cytokines such as IL-6. The unappreciated level of CAF heterogeneity was further highlighted by the Tuveson laboratory with the discovery of another antigen-presenting CAF subtype (apCAFs)^52^. This result suggests the urgent need to dissect the molecular mechanisms driving the complexity of CAFs for the purpose of developing targeted therapies. Another study by the Tuveson laboratory characterized different CAF subtypes by revealing that TGFβ/SMAD2/3 signaling is key in activating myCAFs near tumor cells where the TGFβ gradient is high and that IL1α/JAK/STAT signaling is essential for activating iCAFs in regions where the TGFβ gradient diminishes, illustrating how spatial variations in the TGFβ concentration influence CAF phenotypic differentiation^53^. Recently, a study by Thompson’s group further elucidated the induction of an inflammatory phenotype in CAFs by hypoxia, which stimulated HIF1α-mediated VEGF expression in fibroblasts to support tumor growth^54^.

Building on the foundation of CAF heterogeneity and the dynamic TME of PDAC, PDO models cocultured with various cellular components of the TME have been utilized to study the role of tumor–stroma interactions in tumor progression. While CAFs are widely thought to be educated by cancer cells, whether CAFs play a role in PDAC tumorigenesis remains largely unknown. PDAC initiation is associated with acinar-to-ductal cell metaplasia (ADM), which is the transdifferentiation from the acinar to the ductal phenotype in response to *KRAS* mutation or inflammation. Using an acinar cell organoid model cocultured with CAFs, Seema Parte and colleagues reported the first evidence of a role for CAFs in the induction of ductal transdifferentiation in normal and KRAS-mutant organoids^55^. This CAF-induced ADM was shown to be mediated by LAMA5/ITGA4 crosstalk between CAFs and acinar cells, which possibly stimulates the expression of ductal-specific genes in a STAT3-dependent manner. Additionally, CAFs can support tumor maintenance via the secretion of stem cell niche factors. By establishing 39 PDAC PDOs through niche-based selection, Sato’s group discovered a subtype of Wnt-nonsecreting PDAC that requires Wnt from CAFs for survival and growth^56^. Interestingly, this requirement was dependent on the juxtacrine interaction between PDAC cells and CAFs. The maintenance of cancer stem cells in PDAC through niche factors such as Wnt was recently reported to depend on other TME components, such as endothelial cells. Choi and colleagues showed that the population of CD44^+^ cancer stem cells in PDAC PDOs upon coculture with HUVECs was significantly decreased by Wnt and Notch inhibitors^57^. Moreover, the coculture system also facilitates the study of stroma invasion during PDAC progression. In 2018, Nakamura’s group reported that direct contact between PSCs and PDOs enables cancer cell invasion by destroying the basement membrane^58^. This destruction was caused by the matrix metalloproteinase 2 (MMP2) secreted by PSCs, which was activated by binding to MT1MMP on the PSC membrane.

Not surprisingly, organoid technology also provides a useful platform for investigating the immune response during tumor progression. In 2018, Bishehsari et al. were some of the first to reveal the ability of KRAS-mutated organoids to induce macrophage polarization into the M2 subtype, which, in turn, suppressed the expression of epithelial PEDF via the EGF/EGFR pathway^59^. Another approach by Zavros’s group revealed that the PDAC TME is enriched with polymorphonuclear (PMN) myeloid-derived suppressor cells (MDSCs), which promote tumor growth and suppress cytotoxic T lymphocyte proliferation^60^. The presence of PMN-MDSCs even abrogated the effect of nivolumab on PD-L1-expressing organoids. Moreover, the exploration of immunotherapy holds promise in PDAC treatment, yet patient response rates remain variable. Meng and colleagues primed patient-derived peripheral blood mononuclear cells (PBMCs) with matched PDOs to clonally expand tumor-targeting T cells and identify tumor-targeting T-cell receptors of this cytotoxic T-cell population^61^. They also suggested the involvement of the NKG2A-HLA-E axis, rather than PD-1-PD-L1, as an immune checkpoint for cytotoxic CD8^+^ T cells in PDAC. Cocultures with T cells have also been utilized to study immunomodulatory factors secreted by cancer cells. In a study by Lidström et al., galectin 4 was identified as an important driver of immune evasion in patients with PDAC. Galectin 4 was shown to be secreted by cancer cells and to induce apoptosis in T cells by binding N-glycosylated residues on CD3ε/δ^62^.

Recently, a coculture system of PDOs and macrophages was utilized to elucidate the role of macrophages in the clinical challenge of gemcitabine resistance in PDAC^63^. Briefly, PDAC-secreted AREG stimulates the production of CCL5 from macrophages, which confers stemness and chemoresistance to cancer cells through the CCR5/AKT/Sp1/CD44 axis.

Additionally, D’Angelo and colleagues reported increased T-cell activation, including increased production of granzyme B and the activation marker CD137, when murine PDAC organoids were cocultured with PBMCs isolated from healthy mice, highlighting the potential for preclinical modeling of immunotherapy responses^64^. This approach was further advanced by Zhou and colleagues, who incorporated additional TME components, such as endothelial cells, CAFs, and macrophages, into PDAC organoid cultures^65^. Their studies revealed T-cell infiltration into PDAC organoids, yielding a T-cell-incorporated PDAC organoid model that recapitulates the immunosuppressive TME. Drug screening efforts within this model identified BET and HDAC inhibitors, ITF2357 and I-BET151, as potent enhancers of PDAC antigen presentation and the antitumor activity of cytotoxic T cells, offering a robust platform for discovering immunotherapy vulnerabilities. Further in-depth analysis by Knoblauch and colleagues revealed alterations in T-cell differentiation under coculture conditions, characterized by decreased numbers of CD4+ memory T cells and increased numbers of CD4+ regulatory T cells, indicative of an immunosuppressive environment^66^. Overall, the PDAC organoid and immune cell coculture models, particularly those developed by Zhou and colleagues, faithfully recapitulated the immune TME in vivo, providing a reliable preclinical tool to elucidate the underlying immunotherapy resistance mechanisms and develop effective therapeutic strategies. This precise approach could revolutionize PDAC treatment, shifting from one-size-fits-all therapies to customized therapeutic combinations that are significantly more effective.

**Fig. 3:**
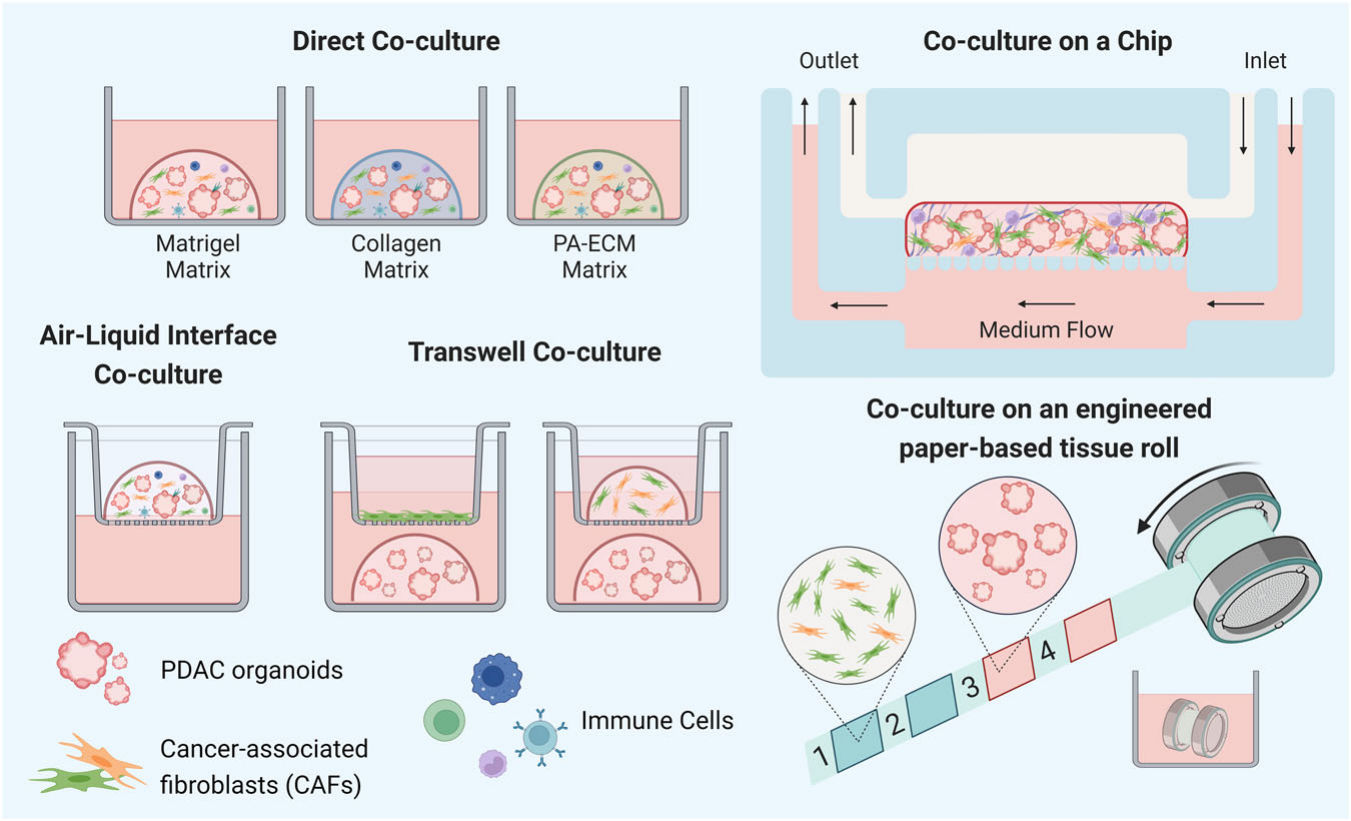
Coculture models of PDAC organoids with TME components. By incorporating various cellular and extracellular components of the TME, the PDAC organoid model serves as a platform to accurately recreate the TME. This model includes the use of different matrices and a variety of coculture methods.

As a result, the coculture system of PDOs, stromal cells, and immune cells is promising for better recapitulating the in vivo TME. Thus, engineering these coculture models provides a more reliable platform for studying drug responses for therapeutic development (Fig. 3). In 2021, Loessner’s group developed a highly tunable coculture system using custom peptide amphiphile ECM components (PA-ECMs) to maintain tumorigenicity with cancer stem cell functionality^67^. This system particularly retains patient-specific transcriptional profiles, recapitulates the PDAC matrisome, and reproduces the response to standard-of-care PDAC treatment (gemcitabine/nab-paclitaxel). Additionally, coculturing PDOs and CAFs has been shown to induce epithelial-to-mesenchymal transition gene signatures in PDAC organoids and confer resistance to chemotherapies, including gemcitabine, 5-FU, and paclitaxel^68,69^. Haque and colleagues transferred this coculture system to a microfluidic chip to better mimic in vivo conditions by incorporating a continuous flow of medium^70^. This PDO-on-a-chip approach also allows drug perfusion to test the ability of anti-stromal agents to augment the PDAC response to gemcitabine. Geyer et al. developed a different microfluidic platform and reported the potential effect of hypoxia on the response of PDAC to gemcitabine treatment in a coculture system^71^. The coculture system can also serve as a system for screening new therapeutic strategies. For example, Machinaga’s group tested the efficacy of an antibody‒drug conjugate (ADC) in delivering a BET protein degrader to the PDAC TME^72^. The authors showed that CEACAM6-targeted ADCs had lethal effects on PDOs and bystander effects on CAFs. This finding was also confirmed in xenograft models, with a decrease in the inflammatory phenotype of CAFs without any significant body weight loss. McGuigan’s group recapitulated the TME in PDAC using an in-house platform, TRACER (tissue roll for analysis of the cellular environment and response), to not only coculture PDOs and PSCs but also mimic the spatial configuration of cells with different oxygen levels in the TME architecture^73^. Briefly, PDOs and PSCs are seeded separately on a strip, which is then rolled to stack the cell layers, recreating tumor heterogeneity in its local architecture with an oxygen gradient during culture. This TRACER platform also allows easy cell isolation and high-dimensional single-cell analytic methods such as CyTOF. Lahusen et al. utilized a standardized agarose microwell chip array system to establish homogenously sized PDO/T-cell/PSC cocultures in a collagen matrix for high-throughput analyses of T-cell infiltration^74^. This model also suggested the potential function of PSCs in inhibiting effector T cells in coculture with PDOs.

Overall, the incorporation of stromal cells and immune cells into the PDO culture system provides a reliable platform for recapitulating the “cold” TME of PDAC, studying tumor–stroma crosstalk, and investigating the immune response upon treatment. Therefore, these coculture models are also expected to revolutionize PDAC therapeutic treatment in its entirety by discovering novel targets for drug development and reproducing all remaining challenges in drug delivery for ex vivo testing.

## Conclusions

The development of pancreatic organoid models has been a cornerstone in the advancement of pancreatic cancer research, providing an innovative and versatile platform that closely mirrors the complexity of in vivo conditions. These models have facilitated critical discoveries across various areas of cancer biology, allowing us to better understand this disease. As highly adaptable preclinical models, organoids facilitate investigations into diagnostic and therapeutic strategies, and provide a robust basis for personalized medicine approaches in clinical settings. While these systems are primarily in vitro/ex vivo, ongoing refinements in organoid technology continue to integrate a broader spectrum of cellular components of the TME. This integration enhances the physiological relevance of these models, enabling more accurate simulations of cellular interactions and drug responses. By improving the incorporation of diverse TME elements, such as immune and stromal cells, pancreatic organoids are poised to provide even more comprehensive insights into the complexities of pancreatic cancer biology, ultimately improving current therapeutic strategies and patient outcomes.
